# Ether lipids and a peroxisomal riddle in sperm

**DOI:** 10.3389/fcell.2023.1166232

**Published:** 2023-05-15

**Authors:** Mayrene Horta Remedios, Weisheng Liang, Lucas N. González, Victoria Li, Vanina G. Da Ros, Débora J. Cohen, Vanina Zaremberg

**Affiliations:** ^1^ Department of Biological Sciences, University of Calgary, Calgary, AB, Canada; ^2^ Instituto de Biología y Medicina Experimental (IByME-CONICET), Buenos Aires, Argentina

**Keywords:** sperm, ether lipid, peroxisome, metabolism, oxidative stress, fertility

## Abstract

Sperm are terminally differentiated cells that lack most of the membranous organelles, resulting in a high abundance of ether glycerolipids found across different species. Ether lipids include plasmalogens, platelet activating factor, GPI-anchors and seminolipid. These lipids play important roles in sperm function and performance, and thus are of special interest as potential fertility markers and therapeutic targets. In the present article, we first review the existing knowledge on the relevance of the different types of ether lipids for sperm production, maturation and function. To further understand ether-lipid metabolism in sperm, we then query available proteomic data from highly purified sperm, and produce a map of metabolic steps retained in these cells. Our analysis pinpoints the presence of a truncated ether lipid biosynthetic pathway that would be competent for the production of precursors through the initial peroxisomal core steps, but devoid of subsequent microsomal enzymes responsible for the final synthesis of all complex ether-lipids. Despite the widely accepted notion that sperm lack peroxisomes, the thorough analysis of published data conducted herein identifies nearly 70% of all known peroxisomal resident proteins as part of the sperm proteome. In view of this, we highlight open questions related to lipid metabolism and possible peroxisomal functions in sperm. We propose a repurposed role for the truncated peroxisomal ether-lipid pathway in detoxification of products from oxidative stress, which is known to critically influence sperm function. The likely presence of a peroxisomal-derived remnant compartment that could act as a sink for toxic fatty alcohols and fatty aldehydes generated by mitochondrial activity is discussed. With this perspective, our review provides a comprehensive metabolic map associated with ether-lipids and peroxisomal-related functions in sperm and offers new insights into potentially relevant antioxidant mechanisms that warrant further research.

## Introduction

**FIGURE 1:**
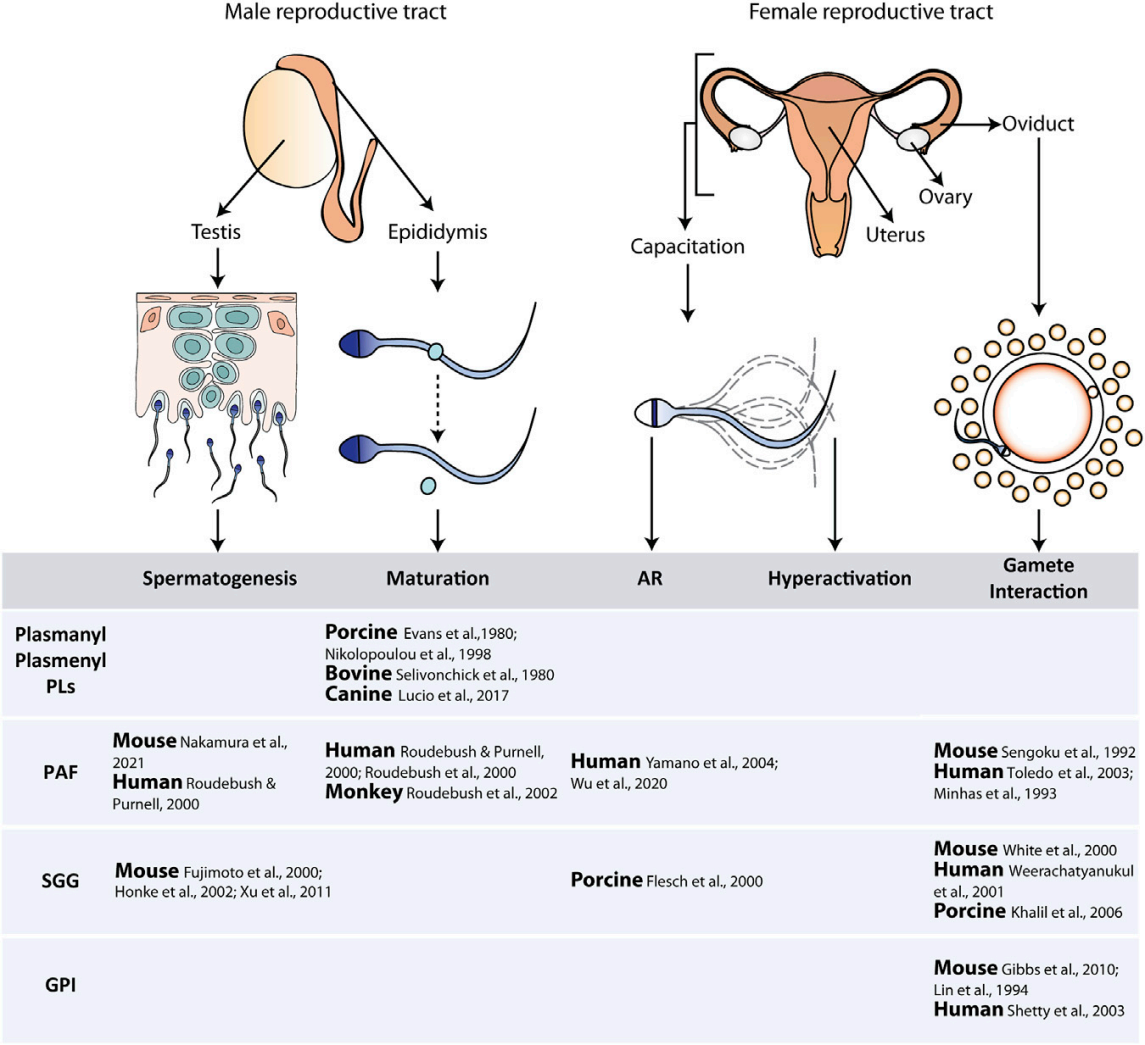
**Schematic representation of the different steps involved in sperm production, maturation, and fertilization.** The described roles for the different types of ether lipids in these steps are included in the table. Abbreviations: AR, acrosomal reaction; PLs, phospholipids; PAF, platelet activating factor; SGG, sulfogalactosylglycerolipid; GPI, glycosylphosphatidylinositol.

Sperm are terminally differentiated haploid cells with a unique structure necessary for the different stages of fertilization and early embryonic development. To achieve fertilization, sperm leaving the testis must first undergo a series of physiological changes in the epididymis and the female tract, known as maturation and capacitation, respectively (Yanagimachi, 1994). As a consequence of these processes, sperm become able to undergo the acrosome reaction, an exocytotic event that takes place in their head, and to develop a specific flagellar movement termed hyperactivation. These physiological changes enable sperm to cross the *cumulus oophorus* that surrounds the egg, then bind to and penetrate the *zona pellucida*, and finally fuse with the egg’s plasma membrane (Figure 1). In this regard, it is important to mention that, since sperm are transcriptionally and translationally silent cells, signal transduction cascades and metabolic pathways, among others, are essential for the regulation of the changes conducive to fertilization. Moreover, sperm function is critically influenced by reactive oxygen species (ROS) mainly derived from aerobic metabolism in the mitochondria. Small quantities of ROS are needed for normal sperm function, stimulating both sperm capacitation and fertilization reviewed by O’Flaherty and Scarlata (2022). However, high levels of ROS induce an oxidative stress state that has a detrimental effect on sperm motility, capacitation, acrosome reaction and DNA integrity, leading to male infertility reviewed by Aitken et al., (2022). As terminally differentiated cells, sperm are particularly vulnerable to ROS attack. Since the late 70’s, evidence has been accumulating showing the critical role of lipid peroxidation in both combating and propagating this damage (Jones and Mann, 1976; Aitken et al., 1989; Gomez et al., 1998; Aitken and Baker, 2006). Therefore, a delicate balance between ROS production and antioxidant mechanisms should exist in normal sperm to ensure sperm survival for successful fertilization.

**FIGURE 2:**
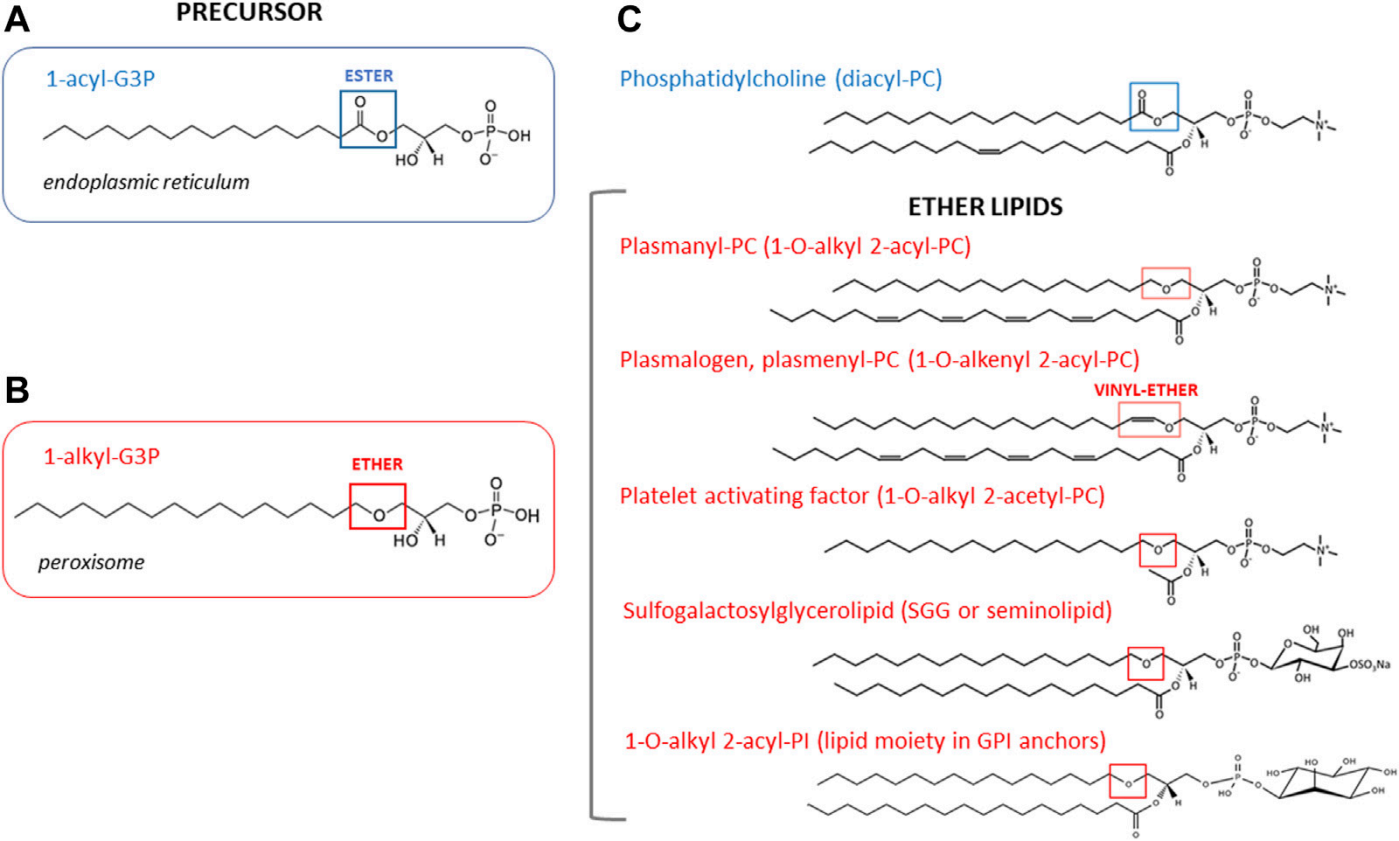
**Chemical structures of ether and diacyl glycerophospholipids and their precursors.** The acyl precursor of diacyl phospholipids, 1-acyl-G3P (also known as lysophosphatidic acid) is shown for comparison. This precursor is mainly made in the endoplasmic reticulum. Blue box points to the ester bond at the *sn-1* position of the glycerol backbone **(A)**. The precursor of all ether lipids is 1-alkyl-glycerol-3-phosphate (1-alkyl-G3P) which is synthesized in peroxisomes. Red box points to ether linkage at the *sn-1* position of the glycerol backbone **(B)**. Structures of representative ether lipid species discussed in this review are shown with the ether linkage boxed in red **(C)**. Note that in the case of plasmenyl glycerophospholipids a vinyl ether bond is found at the *sn-1* position.

As a result of several studies focusing on the evaluation of sperm lipid composition, increased attention has been given to the relationship between sperm lipid composition and fertility (Rivera-Egea et al., 2018; Lopalco et al., 2019). Thus, lipids are of special interest as potential fertility markers and therapeutic targets not only because of their structural functions in sperm, but also because they are sensitive to external and environmental signals, many of which can be recreated *in vitro*. Ether lipids are a particularly highly abundant subclass of glycerophospholipids found in sperm of different species, such as humans, boars, stallions, bulls, and lions (Parks and Lynch, 1992; Leßig et al., 2004; Oresti et al., 2011; Jakop et al., 2022), suggesting they play a critical role in sperm physiology. In other cell types, ether lipids are key components of cell membranes, providing unique structural attributes with effects on membrane dynamics, including membrane fluidity and membrane fusion, facilitating signaling processes, regulating cell differentiation, and protecting membranes from oxidation by acting as potential endogenous antioxidants reviewed by Dean and Lodhi, (2018), Jiménez-Rojo and Riezman, (2019). Structurally, ether-phospholipids are characterized by the presence of an ether bond at the *sn-1* position of the glycerol backbone (Figure 2). Lipids with distinctive ether linkages may be categorized into a variety of subspecies that include: a) plasmanyl and plasmenyl (plasmalogens) glycerophospholipids that compose approximately 20% of the mammalian total phospholipid pool (Dean and Lodhi, 2018), b) platelet-activating factor (PAF) that mediates inflammatory responses (Kelesidis et al., 2015), c) glycosylphosphatidylinositol (GPI) anchors, a lipid anchor for many cell-surface proteins added via posttranslational modifications (Kinoshita, 2016), and d) sulfogalactosylglycerolipid (SGG), also known as seminolipid, which is a testis-specific sulfoglycolipid essential for germ cell function in spermatogenesis (Zhang et al., 2005) (Figure 2). It is not clear when and where this array of ether lipids present in sperm is synthesized. The enzymes responsible for the initial steps in the synthesis of ether lipids are localized to the peroxisome where the alkyl precursor, 1-alkyl-glycerol-3-phosphate (1-alkyl-G3P), is made, and then is transported to the endoplasmic reticulum (ER) and Golgi, where it is consumed to produce complex ether lipids. It is currently accepted that sperm are devoid of these organelles, as these are lost with the residual body and cytoplasmic droplet during spermiogenesis and epididymal maturation, along with other membranous organelles such as lysosomes (Aveldano et al., 1992; Lüers et al., 2006), precluding the idea that they can synthesize ether lipids for themselves. As such, the source of ether lipids in sperm may be linked to early stages of the spermatogenesis or to acquisition during epididymal transit, particularly since the incorporation of exogenous phospholipids is possible through extracellular vesicles such as epididymosomes (Sullivan and Saez, 2013). Intriguingly, reports on sperm proteomes from different species have consistently identified peroxisomal resident proteins, including the enzymes involved in the production of ether lipids (Chauvin et al., 2012; Amaral et al., 2013; 2014; Castillo et al., 2018; Martín-Cano et al., 2020; Greither et al., 2023). Therefore, sperm seem to preserve selected peroxisomal pathways relevant for lipid metabolism. Here, we first summarize existing knowledge on the biological significance of ether lipid species plasmanyl and plasmenyl glycerophospholipids, PAF, SGG and GPI, particularly regarding sperm maturation and fertilization and their impact on male fertility and sperm performance. Using published data from highly purified human and mice sperm, we then embark in a thorough analysis of the sperm proteome identifying peroxisomal resident proteins and ether-lipid metabolic enzymes. This analysis unveils the presence of a truncated biosynthetic pathway where peroxisomal steps capable of producing ether lipid precursors are present, while late ER/Golgi steps are missing. Lastly, we discuss open questions related to lipid metabolism and peroxisomal functions in sperm, which require further research to be answered.

### Ether lipids in the male reproductive system and in sperm

The vast majority of reports on ether lipids in the male reproductive system and sperm date back from two decades ago. The relatively recent identification of genes coding for all the enzymes involved in the biosynthesis of ether lipids (Dean and Lodhi, 2018), combined with powerful techniques using proteomics and lipidomics of sperm samples has revamped research in this field, providing unique opportunities to advance in our knowledge on the role of these relegated lipids highly abundant in sperm.

Examples of the known roles of ether lipids in the different stages of sperm production and function are included in Figure 1.

a) Plasmanyl and plasmenyl phospholipids

During epididymal maturation, the number of phospholipids per cell decreases by around 50% in ram, bull, and rat sperm (Scott et al., 1967; Poulos et al., 1973; Aveldano et al., 1992) due to the loss of the cytoplasmic droplet. Interestingly, this reduction of phospholipids during epididymal transit is selective for diacyl phospholipids whereas plasmalogen levels do not significantly change, resulting in an increase in its proportion. In fact, early lipid analyses have revealed that both plasmanyl (O-) and plasmenyl (P-) forms of phosphatidylcholine and phosphatidylethanolamine are the major phospholipid classes in mature porcine and bovine sperm (Evans et al., 1980; Selivonchick et al., 1980). More recent lipidomics analysis of sperm from humans and other species have confirmed the prevalence of these ether lipids (Wood et al., 2016; Rivera-Egea et al., 2018; Ramal-Sanchez et al., 2020). Although the precise biological function of plasmanyl and plasmenyl glycerophospholipids in the male reproductive system has yet to be elucidated, it is hypothesized that the high proportion of these lipids in mature sperm protects these cells against oxidative damage due to their known antioxidant properties (Shan et al., 2021). Based on their chemical structure, plasmalogens have been considered to act as endogenous antioxidants. Due to their vinyl-ether linkage, plasmalogens are prone to oxidation serving as scavengers for radical species while protecting membrane lipids (Skaff et al., 2008; Broniec et al., 2011). It has been proposed that the susceptibility of plasmalogens to oxidative damage is due to a low hydrogen bond dissociation energy at the carbon adjacent to the vinyl ether bond (Murphy, 2001). Furthermore, the vinyl ether bond might be more exposed to the oxidative agents due to its proximity to the water-lipid interface, making them the first targets of ROS (Lankalapalli et al., 2009). In this regard, oxidation of plasmalogen by interaction with singlet oxygen/ROS occurs significantly faster than their diacyl counterparts (Broniec et al., 2011). However, the formation of further toxic molecules like fatty aldehydes generated from plasmalogens oxidation has also been reported, questioning their role towards a pro-oxidative effect (Stadelmann-Ingrand et al., 2004). This is relevant for sperm, considering the critical effect of ROS, oxidative stress and lipid peroxidation on sperm function, and their high content of polyunsaturated fatty acids incorporated to plasmalogens reviewed by Gibb et al., (2020), Gautier and Aurich, (2022).

Further challenging the idea that all plasmanyl/plasmenyl species play similar roles, is the fact that specific ether lipid species have been linked to opposite phenotypes in sperm performance. For example, 40:4 plasmanyl-PC [(O-40:4)] and 40:5 plasmenyl-PC [(P-40:5)] were identified as potential motility markers in canine spermatozoa (Lucio et al., 2017) while PC (O-42:4) and PE (P-34:2) were found to be significantly higher in sperm from patients without pregnancy success after intracytoplasmic sperm injection (Rivera-Egea et al., 2018). In addition, lack of plasmenyl species did not seem to affect sperm viability (Werner et al., 2020).

In summary, it is possible that ether lipid species with specific length and degree of unsaturation of their fatty acid tails may differentially affect the physical and functional properties of sperm. Indeed, it is known that sperm lipid composition can be influenced by factors such as the intake of dietary lipids (Saez and Drevet, 2019). As such, future investigations may be directed towards establishing a link between ether lipid saturation and alkyl/acyl chain length, the reproductive performance of sperm and its dependence on diet.

b) Platelet activating factor (PAF).

Studies in different species, including humans, have drawn a positive relationship between PAF content and sperm motility (Minhas et al., 1991; 1993; Roudebush et al., 2002), fertilization rates (Toledo et al., 2003) and clinical pregnancy after assisted reproduction treatments (Roudebush and Purnell, 2000). PAF acts through binding to its receptor, which has been found to be concentrated in the proximal head and midpiece of sperm from different species (Reinhardt et al., 1999; Levine et al., 2002). Human sperm samples with less than 50% forward motility possess low PAF receptor levels (Roudebush et al., 2000), leading to the hypothesis that this ether lipid is relevant for sperm function. This has been further supported by the presence of enzymes responsible for PAF catabolism and remodeling in human sperm and seminal plasma (Gujrati et al., 1987).

The association between PAF and sperm function has been studied using experiments where exogenous PAF was added during capacitation. Incubation of mouse sperm in a medium containing PAF resulted in an increase in fertilization rate, while incubation with a PAF receptor antagonist significantly decreased motility and fertilization rate, which was reversed with the addition of PAF (Sengoku et al., 1992). In this regards, PAF-acetylhydrolase, the primary enzyme responsible for inactivating PAF, has been proposed as a decapacitation factor (Letendre et al., 1992; Roudebush and Purnell, 2000; Zhu et al., 2006). More recently, PAF was found to dose-dependently induce the acrosome reaction in capacitated human spermatozoa and has been proposed to be associated with ERK-signaling pathways (Wu et al., 2020).

c) Sulfogalactosylglycerolipid (SGG)

SGG (also known as seminolipid) is an anionic glycolipid found selectively on the outer leaflet of mammalian primary spermatocytes membranes (Ishizuka, 1997; Tanphaichitr et al., 2003). SGG levels reach a maximum in round spermatids and remain constant in sperm (Iwamori et al., 2020; Kongmanas et al., 2021). Saturated C16:0-alkyl-C16:0-acyl is the primary species, with SGG containing other alkyl/acyl chain lengths comprising less than 10% of the total SGG content (Goto-Inoue et al., 2009).

SGG is essential in sperm production as spermatogenesis is arrested in animals lacking SGG synthesis, leading to infertility (Fujimoto et al., 2000; Honke et al., 2002). Moreover, male animals defective in SGG turnover are subfertile (Xu et al., 2011). Altogether, these results support the notion that SGG homeostasis in the testis is critical for normal spermatogenesis.

In addition, SGG involvement in capacitation and fertilization has also been proposed. Several studies have shown that during capacitation, SGG migrates to the equatorial region of the sperm head (Flesch and Gadella, 2000), likely to allow the acrosome reaction to occur. Moreover, most SGG is localized in low-density detergent resistant membranes in capacitated sperm cells, reflecting the ability of SGG to form lipid rafts (Khalil et al., 2006). A role for SGG in sperm-ZP interaction has also been proposed (White et al., 2000; Weerachatyanukul et al., 2001).

In humans, the ratio of cholesterol to SGG is found to be a potential biomarker for semen quality. Oligoasthenozoospermic (i.e., low sperm number and motility) patients were found to have a five-fold higher cholesterol to SGG ratio than individuals with normal sperm motility values, although this ratio has not been implicated to affect fertility (Lopalco et al., 2019).

d) Glycosylphosphatidylinositol (GPI)

GPI is a lipid anchor added post-translationally to many cell-surface proteins (Figure 2). The GPI anchors are assembled on a phosphatidylinositol (PI) lipid in the ER, and then covalently attached to the carboxyl terminus of proteins possessing a GPI-attachment signal peptide (Kinoshita, 2016). In mammals, despite the fact that most free cellular phosphatidylinositols contain unsaturated diacyl glycerol forms, GPI anchors have mainly 1-alkyl-2-acyl phosphatidylinositol with saturated fatty acid tails that make them raft-compatible lipid structures (Kanzawa et al., 2009). GPI anchored proteins (GPI-AP) are typically associated with membrane microdomains or lipid rafts (Kinoshita, 2020).

GPI-APs have been described to play different roles in sperm function. Their origin on the sperm surface is primarily from *de novo* synthesis by spermatogenic cells and/or by transfer from epididymal exosomes (epididymosomes) (Martin-DeLeon, 2015; Yoshitake and Araki, 2020). Database analysis showed more than 25 GPI-APs from testicular origin, and many of them are present in sperm (Yoshitake and Araki, 2020). Among them, TEX101 and Ly6K are essential for male fertility as sperm from knockout mice are unable to reach the fertilization site in the oviduct (Fujihara et al., 2014). Other GPI-APs have been implicated in different steps of gamete interaction (i.e., GLIPR1-like protein 1, SPAM1, SPACA4) (Phelps et al., 1988; Lin et al., 1994; Shetty et al., 2003; Gibbs et al., 2010).

**FIGURE 3:**
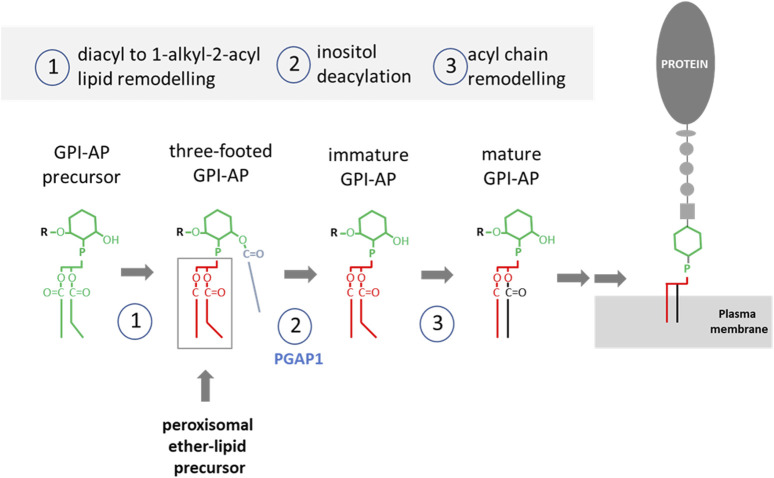
**Key steps in the synthesis of GPI-APs.** The lipid precursor is phosphatidylinositol (PI, green) containing two acyl tails with mostly unsaturated fatty acids in *sn-2*. 1) A diacyl to alkyl/acyl occurs through lipid remodelling involving a peroxisomal precursor. In addition the inositol ring gets palmitoylated. 2) The deacylase PGAP1 removes the acyl tail. 3) Further acyl tail remodeling occurs to introduce a saturated fatty acid in position sn-2. Once mature, the GPI-AP can be delivered to the plasma membrane. Modified from (Kinoshita, 2020).

The key steps in the synthesis of GPI anchors are summarized in Figure 3 (Kinoshita, 2020). GPI-AP synthesis is initiated with a phosphatidylinositol precursor that is converted to a 1-alkyl 2- acyl “three-footed” PI moiety where the inositol ring is acylated in addition to the two alkyl/acyl tails of PI (Kinoshita, 2020). Incorporation of the alkyl-based lipid moiety in the GPI anchor occurs through a remodeling pathway. Although the exact enzymatic steps of this remodeling are unclear, it is known that the peroxisomal alkyl-phospholipid biosynthetic pathway is required for biosynthesis of mammalian GPI anchors (Kanzawa et al., 2009). The inositol ring in the GPI precursor first gets deacylated by the GPI inositol-deacylase Pgap1. Interestingly, Pgap1 has been detected in the proteome of mouse sperm (Chauvin et al., 2012) and PGAP1 knockout mice showed male infertility (Ueda et al., 2007). Therefore, lack of deacylation of the GPI anchor may abolish the subsequent fatty acid remodeling to incorporate the ether lipid moiety, which has been shown to be critical for raft association of GPI-APs (Maeda et al., 2007). This highlights the relevance of alkyl-GPI anchors in male fertility.

### Ether lipid biosynthesis: the core peroxisomal pathway

**FIGURE 4:**
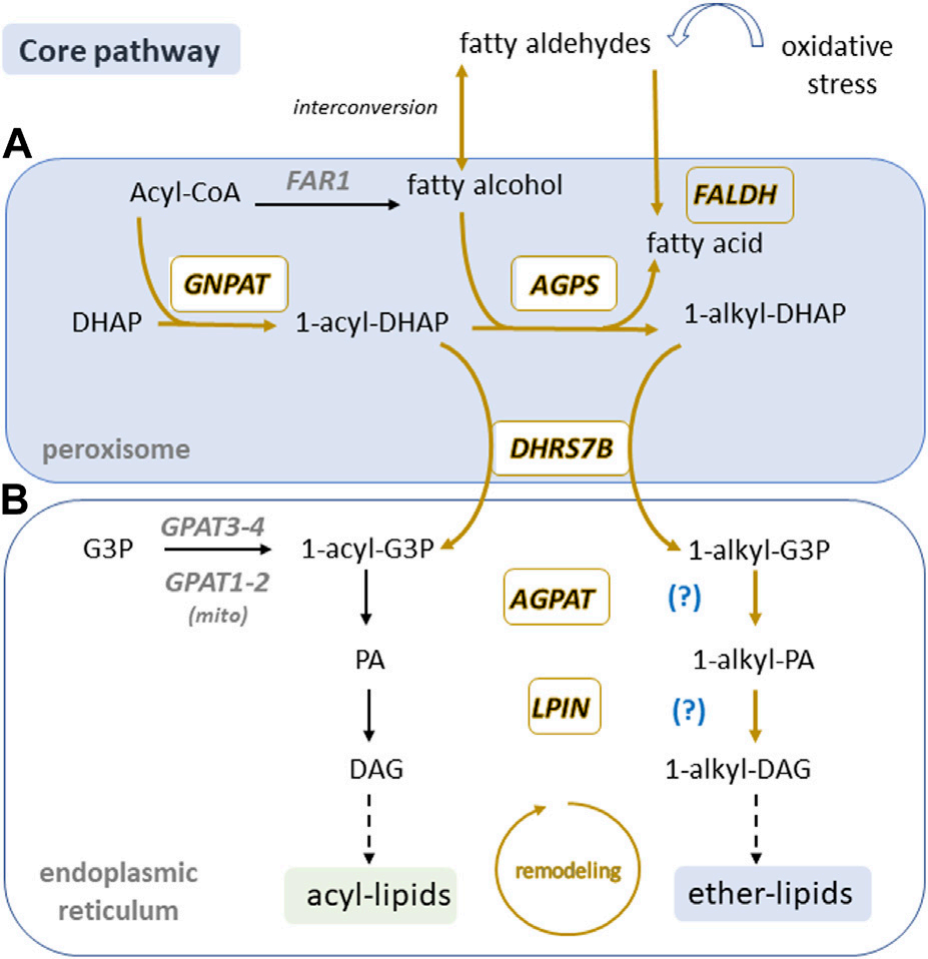
**Peroxisomal core ether lipid biosynthetic pathway is present in sperm.** In somatic cells, ether lipid biosynthesis is initiated in peroxisomes **(A)** and is finalized in the endoplasmic reticulum **(B)** where enzymes may be able to use both acyl and alkyl precursors (question mark). Boxed enzymes and pathways labelled in gold are found in sperm according to Chauvin et al., 2012; Amaral et al., 2013, 2014; Castillo et al., 2018; Martín-Cano et al., 2020 and Greither et al., 2023. Note peroxisome and ER compartments are believed to be missing in sperm. DHAP, Dihydroxyacetone phosphate; G3P, glycerol 3-phosphate; PA, phosphatidic acid; DAG, diacylglycerol; GNPAT, DHAP acyltransferase; AGPS, alkyl-DHAP synthase; DHRS7B, acyl/alkyl DHAP reductase; FAR; fatty acyl-CoA reductase; FALDH fatty aldehyde dehydrogenase; GPAT, G3-P acyltransferase; AGPAT, 1-acyl G3-P acyltransferase; LPIN, lipin PA phosphatase.

The synthesis of all ether lipids is initiated by a core pathway that operates in peroxisomes and is conserved along evolution (Figure 4). In the first step of this pathway, dihydroxyacetone phosphate (DHAP) is acylated at the *sn*-1 position by the enzyme DHAP acyltransferase (GNPAT/DHAPAT) encoded by the mammalian *GNPAT* gene (Dean and Lodhi, 2018). The acyl group on 1-acyl-DHAP is subsequently exchanged for a fatty alcohol via alkyl-DHAP synthase (AGPS/ADHAPS) encoded by the *AGPS* gene. Therefore, this second step is responsible for the characteristic ether bond at the *sn*-1 position, generating the intermediate 1-alkyl-DHAP (Dean and Lodhi, 2018). Studies involving patients deficient in either GNPAT or AGPS revealed a dependence on the presence of the catalytically competent AGPS for the stability and maximal activity of GNPAT (Itzkovitz et al., 2012), leading to the hypothesis that the two enzymes form a functional complex in order to channel 1-acyl-DHAP from GNPAT to AGPS. Both enzymes are localized on the luminal side of the peroxisomal membrane (de Vet E. C. et al., 1997; Thai et al., 1997; Braverman and Moser, 2012) and may exist as a heterotrimeric complex in a 2:1 ratio of GNPAT to AGPS (Biermann et al., 1999; Piano et al., 2016).

The fatty alcohol (AGPS substrate), typically restricted to C16:0, C18:0, and C18:1 species, is generated from a peroxisomal membrane-associated fatty acyl-CoA reductase (FAR), which catalyzes the reduction of acyl-CoA (Cheng and Russell, 2004). Two isoforms, FAR1 and FAR2, with a 58% sequence identity are ubiquitously expressed, albeit at varying levels in different tissues (Cheng and Russell, 2004). FAR1 activity is regulated by the cellular levels of ether lipids as its degradation has been associated with high levels of plasmenyl-PE (Honsho et al., 2010). Therefore, FAR1 is strongly implicated to be the rate-limiting step in ether lipid biosynthesis (Honsho et al., 2013). Another source of fatty alcohols is derived from the interconversion with fatty aldehydes. Evidence supporting the presence of this FAR-independent pathway comes from patients with the inherited metabolic disease Sjögren–Larsson Syndrome (SLS) (Weustenfeld et al., 2019), characterized by a deficiency in the enzyme fatty aldehyde dehydrogenase (FALDH) which oxidizes fatty aldehydes to fatty acids (Keller et al., 2014; Weustenfeld et al., 2019). FALDH deficiency results in an increase of fatty alcohols inducing ether lipid synthesis, reflected by the accumulation of ether lipids in the brain of SLS patients (Staps et al., 2020; Koch et al., 2022).

The last step in the core peroxisomal pathway for ether lipid biosynthesis involves the conversion of 1-alkyl-DHAP to 1-alkyl-glycerol-3 phosphate (1-alkyl-G3P), mediated by an acyl/alkyl DHAP reductase (DHRS7B/ADHAPR) encoded by the *DHRS7B* gene (Lodhi et al., 2012; Honsho et al., 2020). This reductase has the capacity to also reduce 1-acyl-DHAP to 1-acyl-G3P, which is a precursor for the synthesis of phosphatidic acid and all derived acyl-glycerophospholipids (Figure 4). Although the expression of enzymes involved in the previous steps in the pathway are non-tissue specific, *DHRS7B* expression is high in thyroid, muscle, testis, epididymis, and seminal vesicle (Sjöstedt et al., 2020; Human Protein Atlas, 2022). 1-alkyl-G3P is exported from the peroxisome for further acylation and dephosphorylation in the ER, giving rise to the different species of ether lipids discussed above (Honsho et al., 2020).

Peroxisomal disorders associated with ether lipid deficiency typically result in severe clinical phenotypes, highlighting its significance in human pathophysiology. The deficiency of ether lipids could be due to mutations in the biosynthetic peroxisomal proteins (Figure 4) or defects in peroxisomal import complexes. Patients afflicted with Zellweger spectrum disorder lack the ability to assemble functional peroxisomes due to pathogenic variants of *PEX* genes, which encode proteins known as peroxins (Kim and Hettema, 2015). Among these genes are *PEX5* and *PEX7*, coding for cytosolic receptors that recognize **P**eroxisomal **T**argeting **S**ignals, PTS1 or PTS2, respectively, which are necessary for the import of matrix proteins to the peroxisome (Hasan et al., 2013). The core enzymes for the biosynthesis of ether lipids display peroxisomal import signals. Whereas GNPAT contains a PTS1 in its C-end, AGPS presents a PTS2 in its N-end (de Vet E. C. J. M. et al., 1997; Thai et al., 1997; Ofman et al., 1998). A second disorder, rhizomelic chondrodysplasia punctata (RCDP), corresponds specifically to defects in the core ether lipid biosynthesis pathway. Five types of RCDP have been identified; RCDP1 and RCDP5 are associated with defects in the *PEX7* and *PEX5*, respectively, with their abnormal function preventing the import of GNPAT and AGPS to the peroxisomal matrix. Mutations leading to deficiencies in GNPAT, AGPS, and FAR1 are respectively referred to as RCDP2, RCDP3, and RCDP4 (Barøy et al., 2015). Clinical characteristics of RCDP include the shortening of limbs (rhizomelia), premature calcifications of the epiphyseal cartilage, microcephaly, facial dysmorphism, congenital cataracts, and psychomotor retardation (Dean and Lodhi, 2018). Approximately 50% of affected patients succumb by age six due to the severity of these phenotypes, and most do not survive past adolescence (Braverman and Moser, 2012). Studies to elucidate the underlying mechanisms that result in these phenotypes were primarily conducted in knockout mouse models. Mice deficient in GNPAT displayed symptoms observed in RCDP patients, such as dwarfism, cataract formation, and abnormalities in myelination (Rodemer et al., 2003; Teigler et al., 2009). Another symptom of ether lipid deficiency displayed in the mouse models includes male sterility, a characteristic not studied in humans due to the premature mortality associated with the disorder. A targeted disruption of the *GNPAT* gene results in abnormalities of the male reproductive system, including testicular atrophy, decreased seminiferous tubule diameter and absence of sperm in the epididymis (Rodemer et al., 2003). Based on the presence of apoptotic, multinucleated cells within the seminiferous epithelium and a lack of mature sperm or elongating spermatids, a spermatogenic arrest is hypothesized to occur between the pachytene spermatocytes and round spermatid stages (Rodemer et al., 2003). Blind-sterile2 (*bs2*) mice which express aberrantly spliced *AGPS* transcripts exhibited similar phenotypes (Liegel et al., 2011). These observations indicate an essential role of ether lipids in the male reproductive system, raising the question of whether they can serve as valid fertility markers.

### A peroxisomal connection in sperm

As stated before, it is currently accepted that sperm are devoid of peroxisomes, but reports on the proteome of these cells from different species have consistently identified peroxisomal proteins (Chauvin et al., 2012; Amaral et al., 2013; 2014; Castillo et al., 2018; Martín-Cano et al., 2020; Greither et al., 2023). Based on available data on peroxisomal residents at the time, Amaral and others highlighted the presence of fifteen proteins with exclusive peroxisomal localization in the human sperm tail proteome. Furthermore, the expression of peroxisomal membrane protein 11 (PEX11) and peroxisomal 3-ketoacyl-CoA thiolase (ACAA1) was confirmed via immunocytochemistry (PEX11 and ACAA1) and Western blot (ACAA1) in highly purified sperm samples (Amaral et al., 2013). Consistent with MS/MS results using sperm tails, both Pex11 and Acaa1 proteins localized to sperm midpiece and in the case of Acaa1, the full protein was detected by Western blot, indicating it is potentially functional. In order to better define the extent of the peroxisomal presence in the sperm proteome we conducted a meticulous analysis of published proteomic data from human and mouse sperm (Chauvin et al., 2012; Amaral et al., 2013; 2014; Castillo et al., 2018; Greither et al., 2023) and compared it to the known mammalian peroxisomal resident list of proteins (Yifrach et al., 2018). Out of 195 peroxisomal residents, 138 proteins (∼71%) were detected at least once in sperm from humans or mice, representing a much larger incidence than previously anticipated (Supplementary Table S1). It is worth noting that 12 peroxins are present in sperm, with only 4 remaining undetected. Interestingly, two of the missing peroxins (Pex2 and Pex10) belong to the Really Interesting Genes (RING) finger E3 ligases family, and have been recently shown to act as sensors of intracellular FAs, regulating lipolysis in response to ROS levels (Ding et al., 2021). Therefore sperm are equipped with a large set of peroxisomal proteins, with its most abundant matrix proteins (e.g.,.ACOX1, HSD17B4, CAT, THIKA, EPHX2), membrane proteins (ABCD1, ABCD3, ACBD5) and biogenesis associated peroxins in addition to the enzymes from the ether lipid pathway. This probably reflects the presence of a previously undetected peroxisomal remnant compartment which based on previous immunocytochemistry (PEX11 and ACAA1) may be localized to the midpiece in close proximity to mitochondria. Consistently among all sperm proteomes analyzed was the absence of the peroxisomal enzymes FAR1 and FAR2. As highlighted in Figure 4, the core enzymes GNPAT, AGPS and DHRS7B have all been detected in sperm proteomic studies, but the pathway seems to be FAR-independent, suggesting a detrimental role for fatty alcohols in sperm. Our analysis also points to a truncated ether-lipid biosynthetic pathway, with few extra peroxisomal steps present in sperm. A large set of LysoPA acyltransferases (AGPAT1, 2, 3, and 5) and a PA phosphohydrolase (LPIN1), which are the putative enzymes responsible for the production of diacyl- and 1-alkyl-2 acyl- PA and DAG respectively, are also present in sperm (Figure 4). After these steps, the enzymes of the Kennedy pathway known to consume the acyl- and alkyl-DAG intermediates in the ER/Golgi, as well as PEDS, the desaturase that introduces the vinyl bond for the synthesis of plasmalogens (Werner et al., 2020), all seem to be missing from the sperm proteome.

The presence of this truncated ether-lipid biosynthetic pathway suggests a divergent role for the peroxisomal core pathway in sperm. Given the pathway is FAR-independent it must consume an alternative source of fatty-alcohols at the AGPS step. The production of both fatty-aldehydes and fatty-alcohols from the catabolism of membrane lipids in sperm has been documented (Evans et al., 2021). Interestingly, mitochondrial cytochrome c has been found to act as a plasmalogenase that cleaves plasmenylcholine and plasmenylethanolamine at the *sn-1* vinyl ether linkage, releasing fatty aldehydes in response to oxidative stress (Jenkins et al., 2018). The presence of peroxisomal FALDH in sperm also supports a role of a putative peroxisomal-like compartment in the detoxification of fatty aldehydes.

**FIGURE 5:**
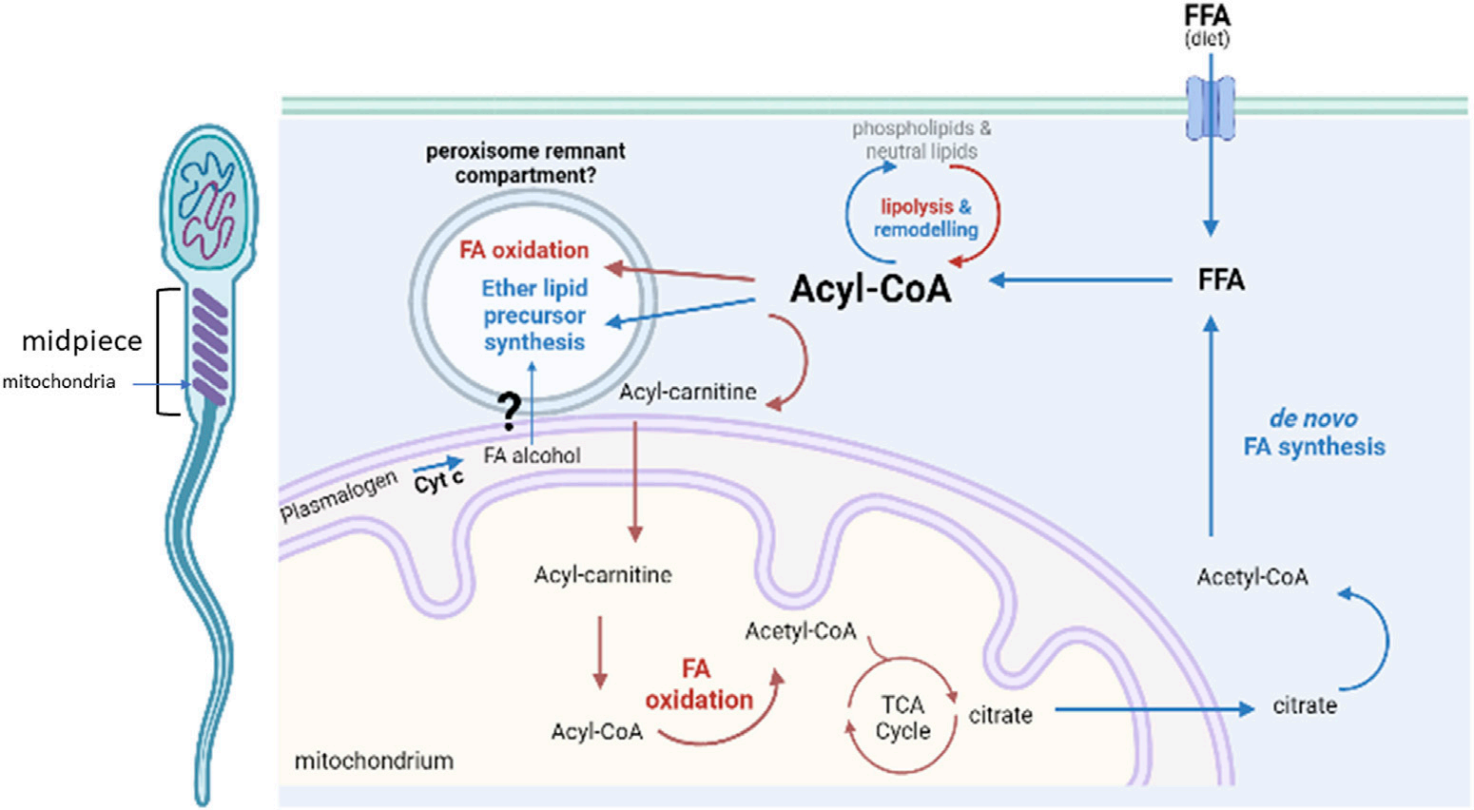
Metabolic lipid pathways present in sperm. Proposed localization of the core peroxisomal pathway to a remnant peroxisomal compartment in close contact with mitochondria, for the synthesis ether lipid precursors with a main fatty alcohol detoxifying role. Abbreviations: Cyt c, cytochrome c; FFA, free fatty acid; FA, fatty acid; TCA, tri-carboxylic acids; CoA, coenzyme A. Created with BioRender.com.

We therefore propose that sperm contain a repurposed FAR-independent ether lipid core pathway, probably localized to a peroxisome-remnant compartment intimately connected to mitochondria in order to act as a sink for toxic fatty alcohols and fatty aldehydes produced from lipid catabolism (Figure 5). Although somehow limited, evidence on the midpiece localization of the peroxisomal residents PEX11 and ACAA1 supports the close proximity of such compartment to mitochondria (Amaral et al., 2013).

In summary, sperm ether lipids, produced in early stages of spermatogenesis and/or provided by the epididymis, play a variety of roles in sperm function included in this review, and probably others to be elucidated in future studies. In addition, this review puts forward the concept that an enhanced collaboration between mitochondria and peroxisomal-like remnant structures may exist in sperm, in order to deal with the oxidative stress burden generated during sperm performance by the high mitochondrial activity (Balbach et al., 2020; Ferreira et al., 2021; Giaccagli et al., 2021).

## Concluding remarks

Ether lipids are particularly abundant in sperm. As we have described in the first sections of this review, their diversity and relevance for sperm production, maturation, and function have been addressed by different laboratories over time. Many outstanding questions remain regarding the regulation of their synthesis and metabolism during the various steps preparing sperm for fertilization. The analysis of available sperm proteomic data carried out in this review identified key lipid metabolic pathways, opening new avenues in this regard. It is well known that sperm damage generated by oxidative stress as a consequence of a systemic inflammation produced by exogenous insults is one of the main causes of male infertility (Agarwal et al., 2018; Su et al., 2022). Therefore, the potential involvement of peroxisomal enzymes in a new antioxidant mechanism to help sperm deal with this damage contributes critical information that has the potential of significantly improving the diagnosis and treatment of male infertility. Future research is needed to challenge the proposed hypothesis and to further investigate the localization of the large peroxisomal subproteome present in sperm.

## Search methods

We performed this study by searching for keywords from PubMed and Web of Science on all articles in English published prior to April 2023. Search terms were based on the following keywords: Ether lipids, Plasmanyl, Plasmenyl, Plasmalogen, Platelet-activating factor, Sulfogalactosylglycerolipid, Seminolipid, Glycosylphosphatidylinositol, GNPAT/DHAPAT, AGPS, DHRS7B, fatty alcohols, oxidative stress, proteomics, and peroxisome combined to male reproductive tract, sperm and fertilization. In all cases, references cited in the analyzed articles were also considered.
